# Supplementary figures and images for: Fine-scale tracking reveals visual field use for predator detection and escape in collective foraging of pigeon flocks
Source: eLife. 2024 Sep 12;13:RP95549. doi: 10.7554/eLife.95549 (PMC11392528; doi:10.7554/eLife.95549)

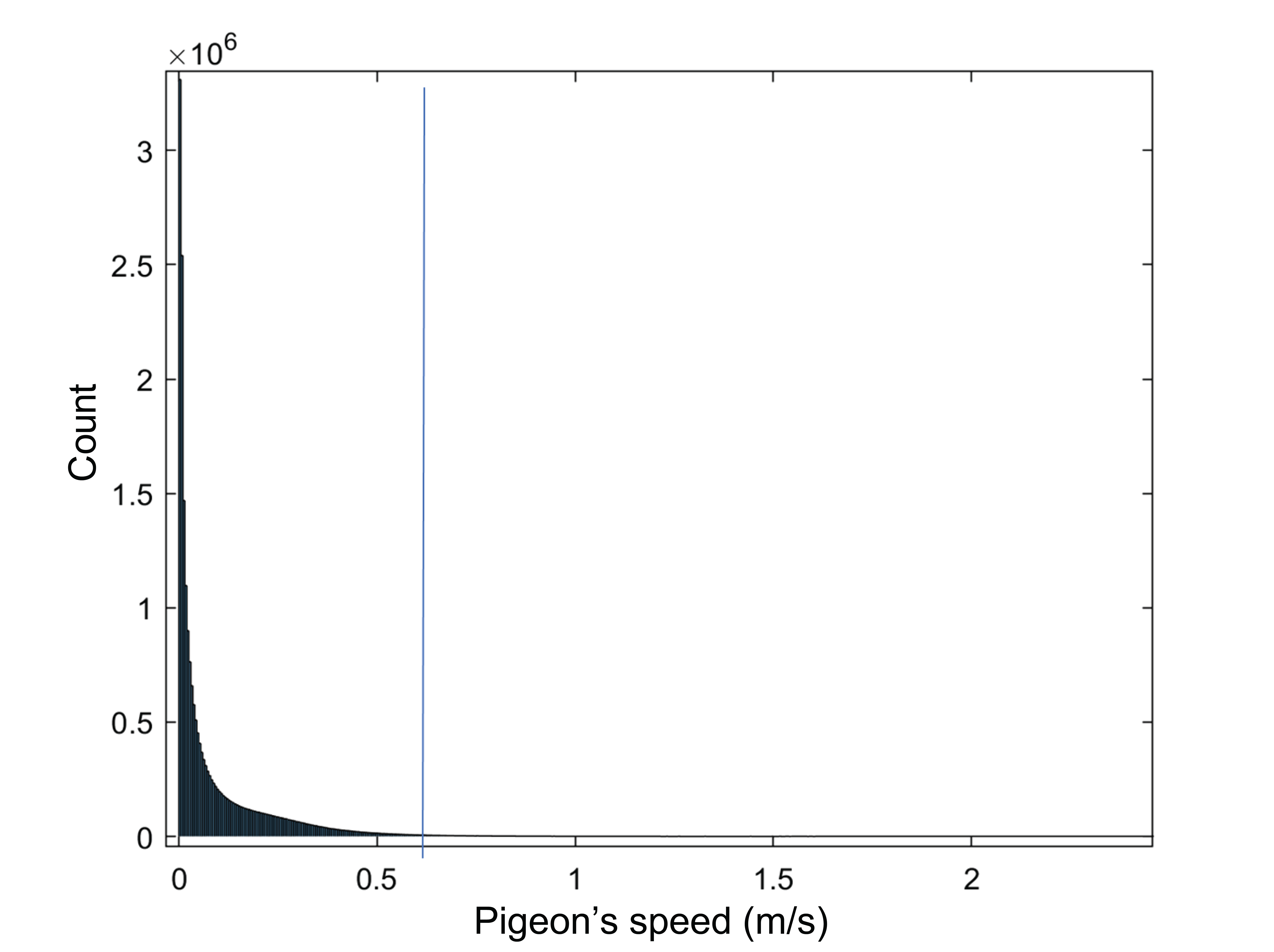

Supplement: Table 2—source data 1. [file elife-95549-table2-data1.zip › Table2-figure_supplement_1.jpg]
